# Longitudinal changes in health-related quality of life according to clinical course among patients with non-tuberculous mycobacterial pulmonary disease: a prospective cohort study

**DOI:** 10.1186/s12890-020-1165-3

**Published:** 2020-05-07

**Authors:** Nakwon Kwak, Sung A Kim, Sun Mi Choi, Jinwoo Lee, Chang-Hoon Lee, Jae-Joon Yim

**Affiliations:** 1 Division of Pulmonary and Critical Care Medicine, Department of Internal Medicine, Seoul National University College of Medicine, 101 Daehak-Ro, Jongno-Gu, Seoul, 110-744 South Korea; 2 Clinical Trials Center, Seoul National University Hospital, Seoul, South Korea

**Keywords:** Health-related quality of life, St. George’s respiratory questionnaire, Non-tuberculous mycobacteria

## Abstract

**Background:**

Improvement in health-related quality of life (HRQL) has been suggested as an alternative treatment goal of non-tuberculous mycobacterial pulmonary disease (NTM-PD). This study was performed to elucidate the longitudinal changes in HRQL using St. George’s Respiratory Questionnaire (SGRQ) among patients with NTM-PD according to their clinical course.

**Methods:**

Patients with NTM-PD who participated in Seoul National University Hospital’s prospective NTM cohort were screened. Participants for whom the SGRQ score was estimated with the one-year interval for ≥ three times were included. The longitudinal trends of the SGRQ score were assessed. The impact of the clinical course on the change in the SGRQ score was elucidated using multilevel mixed-effects linear regression with a repeated-measures model.

**Results:**

In total, 114 patients were analyzed. During the median 5-year observation period, 53 patients started anti-mycobacterial treatment and 61 patients were observed without treatment. Among the treated patients, 24 (45.2%) achieved microbiological cure. Patients who required treatment eventually had worsening SGRQ scores with time compared with patients who could be observed without treatment (*P* < 0.001). In cured patients, the SGRQ score decreased from 33.9 at baseline to 20.8 at 1 year post-treatment (*P* < 0.001), 21.3 at 2 years (P < 0.001), and 17.6 at 3 years (P < 0.001). The SGRQ scores also decreased for 2 years of treatment in patients with NTM-PD that could not be cured, although this decrease did not last for 3 years of treatment.

**Conclusion:**

Worsening HRQL scores were associated with the initiation of treatment and, in turn, treatment improved HRQL scores of patients with NTM-PD.

**Trial registration:**

This study was registered to the ClinicalTrials.gov (Identifier: NCT01616745 / registration date: June 12, 2012). The protocol was retrospectively registered.

## Background

The burden of non-tuberculous mycobacterial pulmonary disease (NTM-PD) has been increasing globally. An increased incidence of NTM-PD has been reported in East Asia [1, 2], the United States [3], and Europe [4]. This phenomenon has also been observed in South Korea. According to a population-based study, the incidence of NTM-PD in South Korea increased from 6.0 cases/100,000 population per year to 19.0 cases/100,000 population per year from 2007 to 2016 [5].

Although the burden of NTM-PD has steadily increased, treatment of NTM-PD is complicated and the outcome is unsatisfactory. Treatment of NTM-PD requires the use of several antibiotics for up to 2 years [6, 7]. However, the treatment success rate was only 60.0% for *Mycobacterium avium* complex (MAC) pulmonary disease [8] and 33.0–41.2% for *M. abscessus* complex pulmonary disease [9–11]. Moreover, microbiologic recurrence is observed among half of patients who have completed treatment for MAC pulmonary disease [12].

Because of the difficulties in eradicating NTM-PD, alternative treatment goals have been suggested. These goals encompass improvements in symptoms, radiographic lesions, and quality of life [6, 13]. In fact, the St. George’s Respiratory Questionnaire (SGRQ) score, which is the most commonly adopted measure of health-related quality of life (HRQL), has been validated for patients with NTM-PD. [14, 15]. The SGRQ score, the decline of which means the improvement of HRQL, was reported to be higher among patients with NTM-PD [16] and has been suggested as an indicator of the treatment response in patients with *M. abscessus* complex pulmonary disease [13]. However, the longitudinal changes in HRQL among patients with NTM-PD have not been fully evaluated. Through this study, we aimed to elucidate the longitudinal changes in HRQL using the SGRQ among patients with NTM-PD according to their clinical course.

## Methods

### Study participants

Patients with NTM-PD who participated in the ongoing prospective Seoul National University Hospital NTM cohort (ClinicalTrials.gov identifier: NCT01616745), which commenced on 1 July 2011, were included in this analysis [17, 18]. The diagnosis of NTM-PD followed the criteria suggested by the American Thoracic Society/Infectious Diseases Society of America [6] and British Thoracic Society guidelines [7]. Patients who completed the SGRQ at least three times with the one-year interval were included in this analysis. Patients with loss to follow-up were excluded. All participants in this cohort were newly diagnosed as having NTM-PD and were treatment-naïve. All patients included in the analysis provided written consents for the study. This study was conducted in accordance with the amended Declaration of Helsinki and the Institutional Review Board of Seoul National University Hospital approved the protocol (IRB No. 1809–112-974).

### Baseline and follow-up evaluations

All patients’ demographic, clinical, and laboratory data were collected and analyzed at the time of study enrollment. Chest computed tomography was performed, and radiographic findings were analyzed. After enrollment, the patients were followed up every 3 to 6 months. On each visit, the participants submitted sputum specimens for acid-fast bacilli smears and mycobacterial cultures and underwent simple chest radiographs. When NTM was isolated from respiratory specimens, the mycobacterial species were identified using the 16S rRNA [19] and *rpo*B gene sequencing [20, 21].

The decision to initiate treatment in each patient was made by the on-duty physicians based on clinical deterioration (e.g., new-onset hemoptysis) or radiographic deterioration defined by two independent chest radiologists (e.g., cavity formation).

### Treatment

The treatment regimens were in accordance with the American Thoracic Society/Infectious Diseases Society of America guideline [6] and were adjusted based on the results of drug susceptibility tests, patients’ tolerance, and adverse events. Generally, patients with MAC infection were treated with a macrolide, rifampicin, and ethambutol. The use of amikacin was determined by the on-duty physicians. Once a decision had been made regarding the use of amikacin, the patients were initially hospitalized for at least 2 to 3 weeks, and the maintenance of injectable drugs was prolonged according to the clinical response and adverse events. For patients with *M. abscessus* complex infection, the combination of antibiotic therapy including a macrolide and at least two parenteral agents including amikacin, cefoxitin, or imipenem were administered as previously described [22]. Once treatment was initiated, the patients were followed up every 4 to 8 weeks.

### SGRQ scoring

The Korean-translated SGRQ, which measures HRQL in patients with diseases of airway obstruction using 76 weighted responses, has been proven reliable and valid for various chronic respiratory diseases [23]. The scores were calculated individually for symptoms, activity, and impacts; the total score was also calculated. The SGRQ was repeatedly performed each year from the time of cohort enrollment among the participants in the present study. The questionnaire performed with an interval of 10 to 14 months were allowed for the analysis. When the patients received antibiotic treatment for NTM-PD, the questionnaire submitted up to 2 months before the time of treatment initiation was adopted for the study.

### Statistical analysis

The patients were categorized into two groups: those who could be observed without treatment and those who needed the treatment for NTM-PD during the follow-up period. The latter group was re-classified into two groups according to the treatment outcomes: 1) microbiological cure, which was defined as three or more consecutive negative and no positive cultures of the causative species from respiratory samples after culture conversion and until the end of anti-mycobacterial treatment [24], and 2) treatment failure, which was defined as the re-emergence of two or more positive cultures or persistence of positive cultures of the causative species from respiratory samples after ≥12 months of anti-mycobacterial treatment, while the patient was still undergoing treatment [24].

The impact of covariates including SGRQ score on treatment initiation was analyzed. In addition, the longitudinal changes in the SGRQ score were compared between the patients who could be observed without treatment and the patients who required treatment later. Once treatment was initiated, the longitudinal trend was also compared between the patients who achieved microbiological cure and the patients who had treatment failure.

Data are expressed as median with interquartile range (IQR) for continuous variables and as proportion for categorical variables. Wilcoxon’s rank-sum test and Fisher’s exact test were used to compare continuous and categorical variables among different groups, respectively. To determine the covariates associated with treatment initiation, multivariate logistic regression egression analysis was adopted. Multilevel mixed-effects regression was used to estimate the effect of each variable on the longitudinal change in the SGRQ score and the predictive means of the SGRQ score at each year. All analyses were performed using STATA version 14.2 (StataCorp, College Station, TX, USA).

## Results

### Patient characteristics

**Fig. 1:**
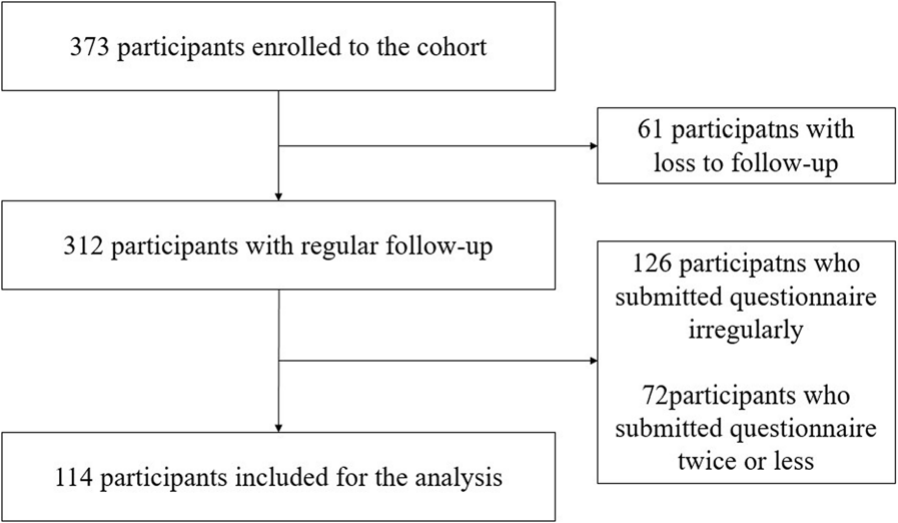
Flow diagram for patients’ inclusion

During the study period, a total of 373 patients was enrolled in the cohort. Sixty one patients who were lost to follow-up, 126 patients who submitted SGRQ questionnaire irregularly, and 72 patients who submitted questionnaire twice or less were excluded for the analysis. Finally, 114 patients and 504 questionnaires were included in the analysis (Fig. 1). Their median age was 63 years (IQR, 57–70 years), and 83 patients were female (72.8%). MAC (74.6%) was the most common pathogen, followed by *M. abscessus* complex (24.6%). The median follow-up duration was 5.0 years (IQR, 4.0–6.0 years). Sixty one patients were regularly monitored without treatment, while 53 patients received anti-mycobacterial treatment. The median interval from enrollment to treatment initiation was 12.0 months (IQR, 2.0–24.0 months). Among the treated patients, 24 (45.3%) achieved microbiologic cure.

The median age, sex proportion, smoking habits, comorbidities, and distribution of etiologic non-tuberculous mycobacteria were not different between patients observed without treatment and those who needed treatment eventually. However, the body mass index (BMI) was higher in patients observed without treatment (*P* = 0.040), while fibrocavitary form was more common in patients who received treatment (*P* = 0.017). Among treated patients, the patients who achieved cure had a higher BMI than those who experienced treatment failure (*P* = 0.024) (Table 1).

**Table 1:** Baseline characteristics of 114 patients with NTM pulmonary disease according to their clinical course

	Patients who were observed without treatment (*n* = 61)	Patients who needed treatment with antibiotics (*n* = 53)	
		Microbiologic cure (*n* = 24)	Treatment failure (*n* = 29)
Age, years	64 (54–72)	61 (54–70)	63 (57–70)
Female sex	40 (65.6)	19 (79.2)	24 (82.8)
BMI, kg/m^2^*	21.4 (19.8–22.9)	21.1 (19.9–22.1)	19.4 (17.8–21.4)
Current/former smoker, n (%)	14 (23.0)	6 (25.0)	4 (13.8)
Comorbidities, n (%)			
History of tuberculosis	23 (37.7)	10 (41.7)	9 (31.0)
Diabetes mellitus	4 (6.6)	2 (8.3)	2 (6.9)
Malignancy	6 (9.8)	3 (12.5)	3 (10.3)
COPD	8 (13.1)	3 (12.5)	2 (6.9)
Radiographic features, n (%)			
Non-cavitary nodular bronchiectatic	51 (83.7)	16 (66.7)	18 (62.1)
Cavitary nodular bronchiectatic	7 (11.5)	3 (12.5)	6 (20.7)
Fibrocavitary	3 (4.8)	5 (20.8)	5 (17.2)
Acid-fast bacilli smear positivity, n (%)	9 (10.7)	7 (5.1)	4 (4.2)
NTM species, n (%)			
*Mycobacterium avium* complex			
*avium*	28 (46.0)	13 (54.2)	9 (31.0)
*intracellulare*	12 (19.7)	8 (33.3)	12 (41.4)
Other *avium complex*^a^	1 (1.6)	1 (3.4)	1 (4.2)
*Mycobacterium abscessus* complex			
Subsp. *abscessus*	13 (21.3)	0	2 (6.9)
Subsp. *massiliense*	5 (8.2)	2 (8.3)	5 (17.2)
Subsp. *bolletii*	1 (1.6)	0	0
*Mycobacterium kansasii*	1 (1.6)	0	0

Data are presented as median (interquartile range) or n (%)

*NTM* non-tuberculous mycobacterial; *BMI* body mass index; *COPD* chronic obstructive pulmonary disease

*BMI was significantly lower in patients who needed treatment than in patients who were observed without treatment (*P* = 0.040). Fibrocavitary form was more prevalent in patients who received treatment (*P* = 0.017). Among patients who were treated, the BMI was significantly lower in patients with failed treatment than in those who achieved cure (*P* = 0.024)

^a^*M. marseillense*, *M. colombiense*, and *M. chimaera* were isolated

### Changes in SGRQ score during observation

**Fig. 2:**
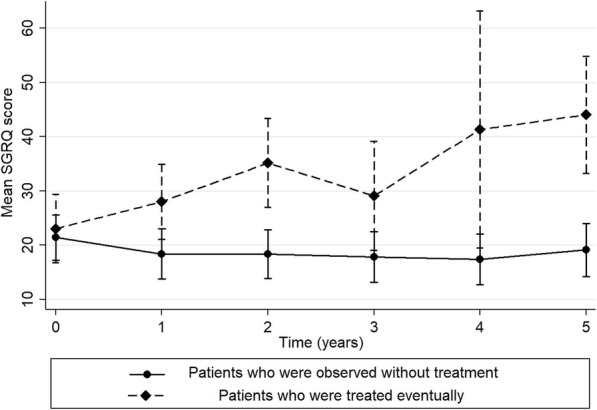
Mean St. George’s Respiratory Questionnaire score with 95% confidence interval over time according to clinical course during observation period

In total, 504 questionnaires were obtained from 114 patients (median, 4 [IQR, 4–5] per patient). When the longitudinal changes of SGRQ scores were compared, patients who eventually required treatment had worsening SGRQ values compared with patients who could be observed without treatment (coefficient, 4.61; *P* < 0.001). While the initial SGRQ scores did not differ between patients who could be observed and patients who eventually required treatment (mean difference, 1.62; 95% confidence interval [CI], − 5.93 to 9.17), the difference between them widened over time (Fig. 2). According to multivariable logistic analysis, changes in SGRQ scores, but not baseline SGRQ, were associated with initiation of treatment (Table 2).

**Table 2:** Association of covariates with treatment initiation

	Adjusted odds ratio [95% confidence interval]	*P*-value
Age (years)	1.66 [0.95, 1.07]	0.789
Female (versus male)	1.01 [0.95, 1.08]	0.430
BMI (kg/m^2^)	0.94 [0.77, 1.15]	0.568
Nodular bronchiectatic form (versus fibrocavitary)	0.79 [0.07, 8.89]	0.851
*Mycobacterium avium* complex (versus others)	1.75 [0.50, 6.11]	0.378
Baseline SGRQ score	1.03 [0.99–1.07]	0.079
Change of SGRQ score (point/year)	1.16 [1.06, 1.27]	0.001

*BMI* body mass index; *SGRQ* St. George’s Respiratory Questionnaire

During the observation period, 17 patients among untreated group achieved spontaneous culture conversion, defined as three or more consecutive negative cultures. However, spontaneous conversion did not affect the changes in SGRQ score (coefficient, − 0.76; *P* = 0.287).

### Changes in SGRQ score with treatment

During the study period, the patients who achieved a microbiological cure received antibiotic treatment with the median duration of 20 (IQR, 18–24) months. Eight of the study patients (five with *M. avium* and three with *M. intracellulare* infection) developed recurrence with the same NTM species. Additionally, one patient with *M. intracellulare* developed an *M. abscessus* subsp. *abscessus* after successful completion of treatment. The patients with treatment failure were treated for the median duration of 24 (IQR, 19–33) months and twelve patients among them were on treatment until the end of the study period. Twelve patients who failed treatment achieved temporary negative conversion of sputum culture followed by re-emergence of multiple positive cultures.

**Fig. 3:**
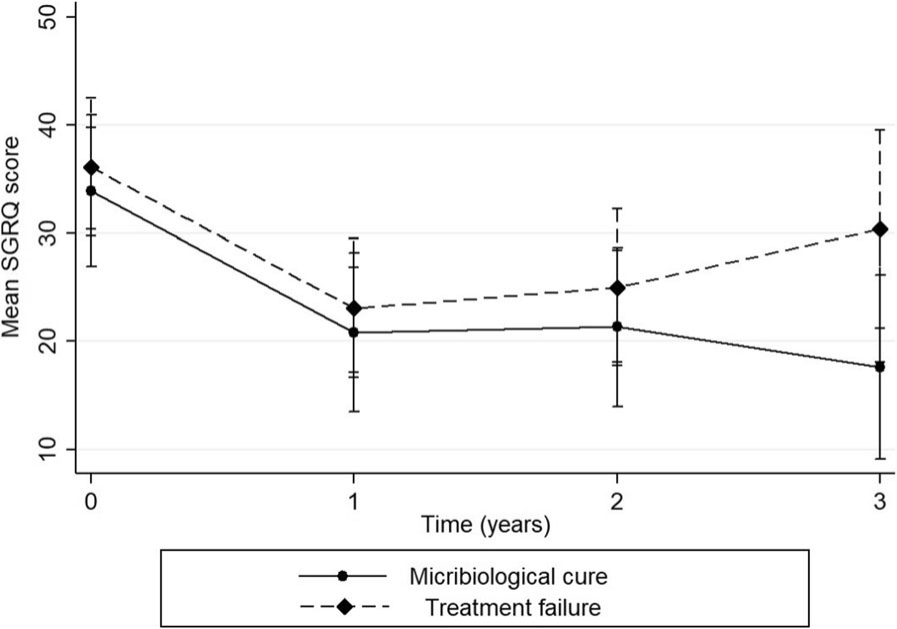
Mean St. George’s Respiratory Questionnaire score with 95% confidence interval over time with treatment

The longitudinal changes of SGRQ score according to the treatment outcomes are illustrated in Fig. 3. Among patients who achieved microbiological cure with treatment, the SGRQ score decreased from 33.9 (95% CI, 26.9–40.9) at the time of treatment initiation to 20.8 (95% CI, 13.5–28.2) (*P* < 0.001) at 1 year of treatment, to 21.3 (95% CI, 14.0–28.7) (P < 0.001) at 2 years, and to 17.6 (95% CI, 9.1–26.1) (P < 0.001) at 3 years. Although patients with treatment failure experienced a decline in the SGRQ score during the first 2 years after initiating treatment (from 36.1 [95% CI, 28.9–42.5] to 23.1 [95% CI, 16.6–29.5] at 1 year and to 25.0 [95% CI, 17.8–32.2] at 2 years), the score at 3 years (30.4) did not decrease. The mean difference at 3 years did not show a significant difference (mean difference, − 5.7 [95% CI, − 14.2 to 2.7]) (Table 3).

**Table 3:** Mean value of St. George’s Respiratory Questionnaire score according to treatment outcomes over time

	Total score		Symptoms score		Activity score		Impacts score	
	Mean (95% CI)	*P*-value	Mean (95% CI)	*P*-value	Mean (95% CI)	*P*-value	Mean (95% CI)	*P*-value
Microbiologic cure								
At treatment initiation	33.9 (26.9–40.9)		47.0 (38.9–55.1)		35.1 (25.4–44.8)		28.5 (20.2–36.8)	
1 year after treatment initiation	20.8 (13.5–28.2)	< 0.001	28.1 (19.4–36.9)	< 0.001	22.8 (12.6–33.1)	0.010	15.7 (7.0–24.5)	0.002
2 years after treatment initiation	21.3 (14.0–28.7)	< 0.001	31.3 (22.8–39.9)	0.001	23.0 (12.9–33.1)	0.009	17.8 (9.1–26.4)	0.008
3 years after treatment initiation	17.6 (9.1–26.1)	< 0.001	31.4 (20.9–41.9)	0.007	21.1 (9.3–33.0)	0.015	17.1 (6.9–27.3)	0.023
Treatment failure								
At treatment initiation	36.1 (29.8–42.5)		51.7 (44.4–59.1)		37.9 (29.1–46.7)		36.1 (28.5–43.6)	
1 year after treatment initiation	23.1 (16.6–29.5)	< 0.001	36.3 (28.8–43.7)	< 0.001	26.9 (18.0–35.8)	0.007	19.0 (11.3–26.6)	< 0.001
2 years after treatment initiation	25.0 (17.8–32.2)	0.001	42.6 (33.9–51.4)	0.054	28.0 (18.0–38.1)	0.035	24.0 (15.4–32.7)	0.003
3 years after treatment initiation	30.4 (21.2–39.5)	0.184	55.0 (43.1–66.8)	0.616	31.5 (18.5–44.5)	0.319	26.8 (15.6–38.0)	0.096

*CI* confidence interval

These trends were also observed for the sub-scores of the SGRQ (symptoms, activity, and psycho-social impacts scores). Although the improvements in the symptoms, activity, and impacts scores were maintained until 3 years of treatment in patients who achieved microbiological cure, these scores returned to the baseline levels at 3 years of treatment in patients with treatment failure. Changes in SGRQ scores over time were not different in the subgroup of patients who had temporary negative conversion of sputum culture but ultimately failed the treatment (coefficient, − 1.06; *P* = 0.873).

## Discussion

We analyzed the longitudinal changes in SGRQ scores according to the clinical course among 114 patients with NTM-PD. While patients who could be observed without treatment showed stationary scores, patients who eventually needed treatment had worsening SGRQ scores during the observational period. Once treatment was initiated, SGRQ scores improved regardless of microbiologic response. However, the improvement did not last among patients who failed in treatment.

The SGRQ was initially developed to measure the health status, including quality of life, in patients with obstructive airway diseases [25]. The SGRQ score could be used as a parameter to assess the treatment responses in patients with bronchiectasis [26] and chronic obstructive pulmonary disease [27]. The SGRQ could also predict the prognosis in patients with chronic obstructive pulmonary disease [28], idiopathic pulmonary fibrosis [29], and chronic pulmonary aspergillosis [30]. Although the HRQL is worse in patients with NTM-PD than in the general population [31], no analysis has been performed to identify the possible association between HRQL and clinical courses of patients with NTM-PD. Our study suggests a role of measuring HRQL using the SGRQ as an additional treatment goal because SGRQ scores can improve even in patients who failed to achieve microbiological cure.

The decision regarding whether to start treatment for NTM-PD has been mainly determined by radiographic or symptomatic changes [6, 7]. However, this decision can be affected by institutions, regions, and nations [32, 33]. The results of this study suggest an association between HRQL and treatment initiation. Although the patients’ age, sex, BMI, radiographic type and NTM species did not affect the initiation of treatment, deterioration of HRQL as measured using the SGRQ was observed in patients who needed treatment eventually. This observation suggests the possibility of a change in the SGRQ score as an indicator of the need for treatment initiation among patients with NTM-PD.

The treatment for NTM-PD is lengthy and complicated, and the treatment outcomes are unsatisfactory regardless of species [8, 10]. In addition, relapse or reinfection after a long duration of treatment has frequently been observed [12, 34]. In this context, a treatment goal other than microbiological cure is required for NTM-PD. In fact, the importance of patient-reported outcomes in future therapeutic trials for NTM-PD has been suggested [35].

Our study showed improvement in HRQL among patients with NTM-PD who were treated. Among patients with NTM-PD cured with treatment, the SGRQ score decreased for 3 years after the initiation of treatment and encompassed all sub-scores of the SGRQ: symptoms, activity, and impact scores. In addition, the improvement in HRQL with treatment was not confined to patients with NTM-PD that had been cured with treatment. Our analysis also showed a decrease in the SGRQ scores for the first 2 years among patients who failed to be cured with treatment. Possible explanation for this is that partial response to treatment, without microbiological cure, could improve patients’ symptoms for a while. These observations suggest that improvement in HRQL could be an alternative treatment goal for patients with NTM-PD.

Another issue related to the treatment of NTM-PD is prediction of which patients need and will benefit from antibiotic treatment [35] because antibiotics are associated with the potential for adverse events and the treatment outcome is unsatisfactory [36]. However, reliable indicators of which patients will benefit from therapy are still lacking [35]. This study showed that even in the patients who failed to be cured, HRQL improved for at least 2 years after the initiation of treatment. This observation suggests that active adoption of treatment should be considered among patients with NTM-PD, especially those with worsening HRQL over time.

Our study has several limitations. First, the number of patients with NTM-PD included in this analysis was relatively small. Only one-third of the patients enrolled in our prospective cohort study could be included in this analysis because the others had only one or two yearly SGRQ scores. Second, SGRQ mainly focused on respiratory symptoms and could not reflect the NTM-specific symptoms such as loss of appetite or memory disturbance, which are measured in ‘NTM symptom module’ [35]. However, most of NTM-PD patients complained of respiratory symptoms [22] and the importance of SGRQ should not be ignored. Third, yearly assessment of SGRQ in this study could dampen the variation of HRQL during the observation period. However, as our study revealed, most of the patients were followed up for a long period and the annual assessment might be more appropriate in real clinical practice.

## Conclusions

In conclusion, worsening HRQL scores were associated with initiation of treatment and, in turn, treatment improved HRQL scores of patients with NTM-PD.

## Data Availability

The dataset used are available from the corresponding author on reasonable request.

## Abbreviations

BMI: Body mass index
HRQL: Health-related quality of life
IQR: Interquartile range
MAC: *Mycobacterium avium* complex
NTM-PD: Non-tuberculous mycobacterial pulmonary disease
SGRQ: St. George’s Respiratory Questionnaire
